# SEnviro: A Sensorized Platform Proposal Using Open Hardware and Open Standards

**DOI:** 10.3390/s150305555

**Published:** 2015-03-06

**Authors:** Sergio Trilles, Alejandro Luján, Óscar Belmonte, Raúl Montoliu, Joaquín Torres-Sospedra, Joaquín Huerta

**Affiliations:** Institute of New Imaging Technologies, Universitat Jaume I, Av. Vicente Sos Baynat s/n, 12071, Castellón de la Plana, Spain; E-mails: alujan@uji.es (A.L.); belfern@uji.es (Ó.B.); montoliu@uji.es (R.M.); jtorres@uji.es (J.T.-S.); huerta@uji.es (J.H.)

**Keywords:** wireless sensor networks, Internet of Things, Web of Things, sensorized platform, open hardware, interoperability, OGC SensorThings API

## Abstract

The need for constant monitoring of environmental conditions has produced an increase in the development of wireless sensor networks (WSN). The drive towards smart cities has produced the need for smart sensors to be able to monitor what is happening in our cities. This, combined with the decrease in hardware component prices and the increase in the popularity of open hardware, has favored the deployment of sensor networks based on open hardware. The new trends in Internet Protocol (IP) communication between sensor nodes allow sensor access via the Internet, turning them into smart objects (Internet of Things and Web of Things). Currently, WSNs provide data in different formats. There is a lack of communication protocol standardization, which turns into interoperability issues when connecting different sensor networks or even when connecting different sensor nodes within the same network. This work presents a sensorized platform proposal that adheres to the principles of the Internet of Things and the Web of Things. Wireless sensor nodes were built using open hardware solutions, and communications rely on the HTTP/IP Internet protocols. The Open Geospatial Consortium (OGC) SensorThings API candidate standard was used as a neutral format to avoid interoperability issues. An environmental WSN developed following the proposed architecture was built as a proof of concept. Details on how to build each node and a study regarding energy concerns are presented.

## 1. Introduction

Nowadays, one of the challenges of our society is to know what is happening around us at every moment and how this affects our daily lives. The growing concerns about climate change, natural disasters, global warming or disease outbreaks make environmental monitoring an important aspect in developed and developing countries [1]. For all of these reasons, there is an increasing demand for the deployment of wireless sensor networks (WSNs) that provide updated information about the state of the environment. Furthermore, the new approach towards the Internet of Things (IoT) [2] offers the possibility to create smart objects and to form WSNs with them.

The trend in hardware manufacturing is to increase the processing capabilities of microprocessors, following Moore's law, while reducing their size. A second important factor is the reduction in price of these devices. This has allowed the use of this technology in many fields where it could not be applied before. The low cost of the technology that forms the sensors has facilitated the proliferation of WSNs in many scenarios, such as environmental monitoring, agriculture, health or smart cities.

A third key aspect that has contributed to the increase in the use of these types of sensors is the open hardware movement. In recent years, there have been several projects that have released the schematics of their devices, which has increased their use. One remarkable example is the Arduino project [3], a low-cost and easy to use microcontroller platform, with a huge community of developers that share information, experiences and knowledge.

A fourth trend is to use Internet Protocol (IP) to achieve connectivity between WSNs and the Internet [4]. The sensors are interconnected to make a WSN, mainly based on open standards, in which each device has its own IP address. In this way, the sensors may be considered as smart objects, which are interconnected in order to make an IoT [2]. IoT describes a concept in which the world of real, physical things is integrated into the virtual world of bits and bytes. This term was first used in a paper by David Brock in 2001 [5]. The term WoT describes the evolution of the IoT [6] and the integration of web standards [7] into this concept.

Within this context, we present our work, which consists of a sensorized platform that can be used to study different kinds of phenomena for multiple uses, like environmental, smart cities, health, and so forth. This platform has been named *Sense Our Environment*(*SEnviro*are the italics necessary? please check throughout ), which is a low-cost and autonomous solution. Each node of the SEnviro platform, called *SEnviro Thing*, belongs to a WSN according to the paradigm of IoT. In order to validate our proposal, a WSN is created using the *SEnviro* approach to monitor the environment. This WSN is deployed and evaluated in the context of Jaume I University's (http://www.uji.es/) campus, which, with a 176,000-m^2^ centralized campus, is a real-life testing scenario for smart city and environmental monitoring developments.

In summary, the main contributions of this work are: (1) a sensorized platform proposal able to make observations in several scenarios, such as environmental, smart cities, health, agricultural, and so on; (2) a low-cost, energetically autonomous and open solution using open hardware and open software; (3) to follow the IoT paradigms and to offer IP connection with agile access; and (4) to provide interoperable access to sensor data by means of the SensorThings API [8], which is an Open Geospatial Consortium (OGC) proposal to work with objects in the IoT paradigm.

The remainder of the paper is organized as follows. Section 2 presents the background of the new trends in WSNs, open hardware and OGC standards. Section 3 presents the architecture proposal of the *SEnviro* platform. Section 4 details the proof of concept of our architecture proposal. Section 5 reviews the related work. The paper concludes in Section 6 with conclusions and future work.

## 2. Background

In this section, we first present the new concepts that the traditional WSNs have added in the last few years and which have evolved into the current state of WSNs. Then, we discuss the current alternatives for WSN development using open hardware. Finally, we detail the standards used for handling sensor data.

### 2.1. Wireless Sensor Networks

A WSN is composed of sensors called nodes. They perform observations and transfer them to another node; this may be a final or an intermediate node. According to [9], there are four different kinds of applications for WSN: data collection, monitoring, surveillance and medical telemetry.

As in traditional networks, WSN have different topologies, such as star, mesh or cluster tree [10]. In order to build these topologies, different components should be defined within the same network. Besides the nodes, there are different parts, which are the router and coordinator [11]. The router exchanges the observations between devices, and the coordinator has control over the network.

WSNs are characterized by their high heterogeneity, as they use lots of proprietary and non-proprietary solutions. Traditionally, WSNs are based on proprietary protocols and No-Internet Protocol (No-IP), such as ZigBee [12], Z-wabe [13], Insteon [14], and so on. One of the most important challenges in this area is to provide interoperability between different WSNs with different protocols [15]. The new WSNs have assimilated other technologies [16], such as Bluetooth, radio frequency identification (RFID), wireless fidelity (Wi-Fi), mobile data services, etc. This implies a direct connection to the sensor, in order to obtain immediate measurements. Furthermore, the sensors have the ability to interact with other sensors in the same network. This allows several strategies of work.

In this way, existing web protocols are used as a common language for communication between different nodes. The Hypertext Transfer Protocol (HTTP) is used as the application layer instead of the transport layer commonly used in web services. Each sensor can be accessed by its Uniform Resource Identifier (URI), and its functionality can be accessed through HTTP known operations (GET, PUT, POST and DELETE). The major benefits of HTTP in WSNs is that it enables the use of standard web services based on the REST architectural style. In addition, applications supporting RESTful services perform better on WSN with limited computational resources [17].

In [18], a proposal to use the REST paradigm with smart objects is presented. According to [6], each sensor can be considered an intelligent object that is able to sense, communicate and act, so each sensor has memory and intelligence. Each sensor is identified by means of a unique URI; in this way, it can be accessed by following the principles of the IoT and WoT [7]. There are two methods for accessing WSN when using a RESTful interface; direct and indirect [7]. In the case of direct access, each sensor is connected directly to the Internet. Indirect access uses a private network that performs a gateway function.

### 2.2. Open Hardware

As already mentioned, the cost reduction and the increase of open hardware popularity have opened up different options for microcontroller-based platforms [19]. Currently, there are several different alternatives regarding the microcontroller-based platform, and the most remarkable platforms are: Arduino [3], Raspberry Pi [20], BeagleBone [21] or MSP430 Launchpad [22]. These options are completely or partially open hardware. Below, we provide a short description of each of them. Table 1 shows a more detailed comparison.

- Arduino: This is a completely open hardware platform designed for reading data from its inputs, processing small volumes of data and producing an output. There are a variety of sensors and actuators that are compatible with Arduino. The main advantage over other platforms is the large amount of resources available, both in terms of software and hardware. Another advantage is its low energy consumption due to its limited processing capabilities. Arduino is the most popular platform and is used in many applications. There are different versions of the Arduino platform with different features.
- Raspberry Pi: This is a partially open hardware platform, which is designed to provide more processing power than Arduino, as it is focused on multimedia due to the HDMI connector. However, you can also add sensors or actuators via its general-purpose input/output (GPIO) pins. Raspberry Pi runs Linux as the operating system, which encourages the development of applications. This platform is more expensive compared to Arduino UNO and has higher energy consumption.
- BeagleBone: This is a completely open hardware platform and is less popular than the former platforms. This platform is very similar to Raspberry Pi. They share many characteristics, both in terms of processing and I/O. Both BeagleBone and Raspberry Pi have been built to have a higher level of abstraction.
- MSP430 Launchpad: This is a hardware platform that is more similar to Arduino, as its features are more limited than Raspberry Pi or BeagleBone. MSP430, as well as Arduino are designed for low-power applications. Unlike Arduino, MSP430 does not have a large community behind it.

For the presented proof of concept (Section 4), we have chosen the Arduino UNO microcontroller. When compared with other platforms, such as the Raspberry Pi and BeagleBone platforms, Arduino UNO is more appropriate for working autonomously, because of its lower energy consumption. Moreover, the monitoring of the environment functionality that we want for our project does not need high processing power. Another reason for the use of Arduino, unlike MSP430, is that it has a lot of expansion cards (shields), which facilitates its use and development. An example of this is the Grove shield . The Grove shield facilitates the connection between sensors and Arduino through a plug-and-play connection. Arduino has a very active developer community, as well.

### 2.3. OGC Standards

A standard-compliant system can be easily reused, because it provides an interoperable communication method. One of the main organizations working in the standardization of WSN is the Open Geospatial Consortium (OGC). The OGC has established Sensor Web Enablement (SWE) as a set of specifications related to sensors, sensor data models and sensor web services that will enable sensors to be accessible and controllable via the web [23]. In order to standardize sensor information, the SWE group offers mechanisms that improve the discovery and access to this data type. The core suite of language and service interface specifications includes the following: observations and measurements (O&M), SensorML, sensor observation service (SOS), Transducer Model Language (TransducerML), sensor planning service (SPS), sensor alert service (SAS), web notification services (WNSs).

The SWE standards enable all sensors to be discovered, accessed and reused via the web. However, SWE standards are as complex as needed, supporting tasks, like controlling Earth imaging satellites. Thus, they are too “heavy” for running applications on devices with limited resources [24].

On the contrary, the OGC SensorThings API [8] can be considered a lightweight SWE profile, particularly well suited for developing WoT applications. The SensorThings API is a standard candidate that provides open access built on web protocols, based on the current SWE and following the architectural REST style. Its aim is to provide a standardized way to expose the real world to the world of the IoT, where things have limited resources.

OGC SensorThings API consists of two layers of standards for connecting various types of WoT sensing devices. The one layer is the IoT resources model layer that enables the understanding and use of heterogeneous IoT devices. This layer consists of the standard-based data model describing the entities and their relationships. The second one is the IoT service interface layer, which defines the URI patterns for WoT resource addressing, the CRUD (create, read, update and delete) operations that WoT resources are able to perform and the query parameters for filtering IoT resources.

In order to provide a common pattern to access the data and capabilities of IoT devices, OGC SensorThings API defines a data model where the core *Entit*yis a *Thing*. Every *Thing* can have zero or more locations in space or time. Furthermore, each *Thing* can have zero or more data streams (which belong to the core of the sensing datastream profile). The data model contains two profiles. A profile is a part of the data model that defines an environment. These profiles are:

- Sensing profile: allows IoT devices and applications to define CRUD data operations in the OGC SensorThings API service.
- Tasking profile: allows applications to control IoT devices through an OGC SensorThings API service.

**Figure 1.**
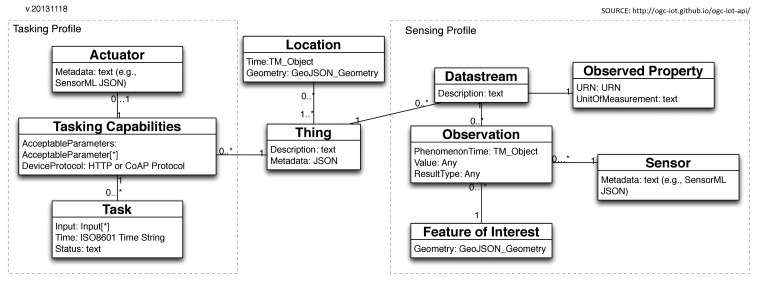
OGC SensorThings API data motel.

The OGC SensorThings API data model is shown in Figure 1. Each *Thing* has a *Location* in space and time. It can have multiple *Datastreams*, which are collections of *Observation* entities grouped by the same *Observed Property* An. *Observation* is an event executed by a *Sensor*, which produces a result whose value is an estimate of the *Observed Property* in the *Feature of Interest*. A *Thing* can have multiple *Tasking Capabilities*, such as an executable function that is executed by an *Actuator*. User can create any number of entity *Tasks* to be run in the service.

**Figure 2.**
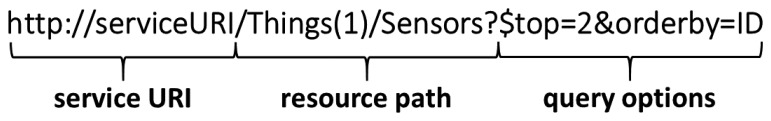
OGC SensorThings API URI pattern.

Figure 2 shows the three components defined by a REST URI: the root URI, resource path and query options. The URI is the location of the OGC SensorThings API service. By attaching the resource path after the root URI service, users can select any resources available in an OGC SensorThings API service. When users perform a read action on a resource, some query options could be provided, such as sorting or filtering with different criteria. We will use OGC SensorThings API as the interface to describe the observations provided by the *SEnviro* network.

## 3. SEnviro Architecture Proposal

In this section, we first present a general overview of the sensorized platform. Secondly, we present the conceptual hardware design of the *SEnviro Thing*. Thirdly, the behavior of each *SEnviro Thing* is described. Finally, the details of the interface used to fulfil the WoT paradigm and interoperability objectives are presented.

### 3.1. General Overview

The *SEnviro* platform aims to provide a sensorized platform following the IoT and WoT paradigms by means of a low-cost, open, energetically autonomous and interoperable solution. In this way, the *SEnviro* platform introduces a new design proposal to easily attach different kinds of sensors. The *SEnviro* platform uses an IP protocol to establish the connection. With these features, it can be considered that each *SEnviro Thing* is a smart object. A *SEnviro* network is formed by joining several *SEnviro Things*.

The proposed platform is inexpensive, because we have chosen affordable components and sensors. The *SEnviro* platform can be considered open, because we use both open hardware and open software. Another important feature of this platform is that it is energetically autonomous by means of a battery cell, which is charged with the energy provided by a solar panel attached to it. The *SEnviro* platform also offers an interoperable service using standards.

An example realization of our proposal will be fully described in Section 4. As an example, a *SEnviro Thing* can be considered as a hardware and software platform able to integrate any type of sensor and communicate the sensor measures for later storage, processing and analysis; as energy autonomous, highly reconfigurable and fully interoperable. For this first proof of concept, we have added environmental sensors, such as temperature, humidity and CO_2_, among others.

### 3.2. SEnviro Things Design

**Figure 3.**
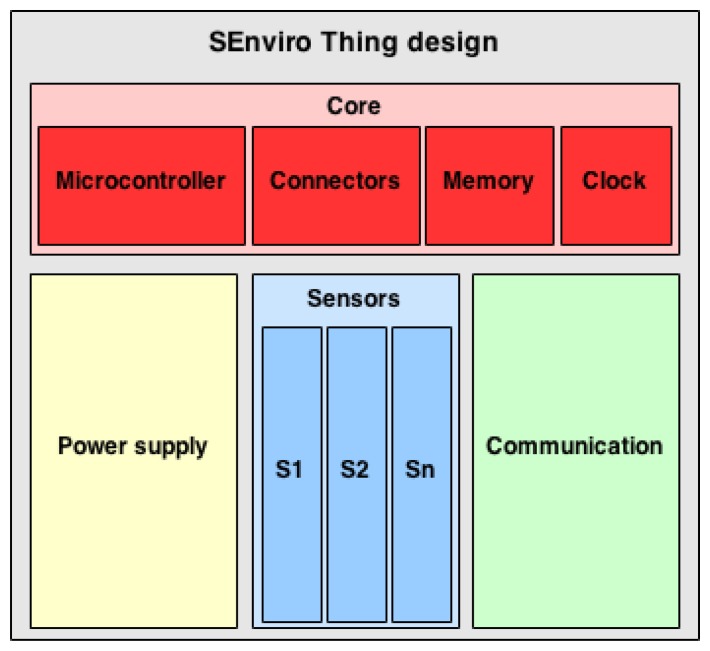
*SEnviro Thing* design.

The *SEnviro Thing* has been designed to be a node acting as a smart object, which provides environmental measurements. Each *SEnviro Thing* is formed by different components, which are organized into four groups depending on their function: *Core, Sensors, Power Supply* and *Communication* (Figure 3).

The *Core* of the system collects, stores, sends and manages the sensor data. The *Core* structure of the system also provides the hardware interfaces and suitable communication protocols needed to connect it with the sensors. To fulfil these objectives, the *Core* is formed by four parts: *Microcontroller*, *Connectors, Clock and Memory*.

- The *Microcontroller* is the most important component in the *Core*. It processes the sensor data and triggers any interaction with either system components or clients.
- The *Connector* module helps to easily and quickly connect the *Core* and *Sensors* parts of the *SEnviro Thing*. This module provides different kinds of interfaces to guarantee the interoperability between the *Sensors* and the *Core* modules.
- Furthermore, each *SEnviro Thing* includes data storage that is used for several purposes. The main purpose is that the *SEnviro Thing* needs to know the current state, which is stored in the *Memory*. Moreover, the *Memory* can be used to save different kinds of data, like historical sensor data.
- Each *SEnviro Thing* needs to add a time-stamp to each measurement for a later analysis of the data. For this purpose, a *Clock* has been included in the system.

The *Core* provides the appropriate basis to build a system where *Sensors* can be attached or detached depending on the particular phenomena that is measured in each particular case. As already stated, the *Core* offers interoperable connectors, which facilitate plugging and unplugging the sensors of the platform, regardless of the nature of the measured phenomenon.

In order to connect with other *Things*, a *Communication* module has been included. Each *SEnviro Thing* must use a communication channel and, thus, suitable communication hardware that provides the *Core* interface to send and receive data. This module offers different types of communication to easily exchange data between *SEnviro Things*.

To offer an autonomous *SEnviro Thing*, a battery and solar panel have been included to provide power supply.

### 3.3. SEnviro Thing Behavior

**Figure 4.**
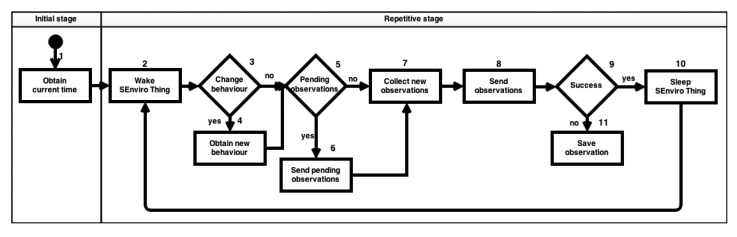
*SEnviro* Thing behavior diagram.

*SEnviro* Thing has been designed to be modular. It provides a core functionality, where users can easily add or remove hardware modules. In this way, it can be ensured that *SEnviro Thing* will continue working whenever a new sensor is added or an existing one is removed. Therefore, *SEnviro Things* is very versatile, as they can be easily adapted to different scenarios and different study proposals. *SEnviro Thing* is able to change its behavior, updating the information about what sensor is active and the frequency of the measurement. Figure 4 shows the defined workflow of a *SEnviro Thing*.

The *SEnviro Thing* behavior has two stages, *initial* and *repetitive*. In the *initial* stage, a set of procedures are executed when it is initiated, for instance one procedure to initialize the clock (Figure 4, Step 1) using the current time. The *repetitive* stage defines the procedures that should be repeated during the *SEnviro Thing's* life cycle. This can be divided into five different steps as follows: (1) the *SEnviro Thing* wakes up from a sleeping period to start collecting data (Figure 4, Step 2); (2) it checks whether to modify their behavior (Figure 4, Step 3); if affirmative, its behavior is changed (Figure 4, Step 4); (3) it checks if there are pending observations in the memory that have not previously been sent (Figure 4, Step 5); if there exist such observations, it sends them (Figure 4, Step 6); (4) it collects new observations from each sensor (Figure 4, Step 7) and tries to send them (Figure 4, Step 8); and (5) after trying to send (Figure 4, Step 9) the data, it goes to sleep (Figure 4, Step 10). If there is any problem sending the data, it saves the data to the internal memory (Figure 4, Step 11) before going to sleep. These data will be sent in the next cycle. Therefore, the *SEnviro Thing's* life cycle starts again with the wake up (Figure 4, Step 2).

### 3.4. Interface for IoT: OGC SensorThings API

One of the objectives of the *SEnviro* platform is to offer a standard service, in order to provide connectivity in an interoperable way. To meet this challenge, an OGC SensorThings API has been used to offer an interoperable service to access the *SEnviro* platform. As previously commented, one of the keys of this standard is that it breaks with all “standard topics” [25] and offers access to restrictive devices, such as smartphones.

**Figure 5.**
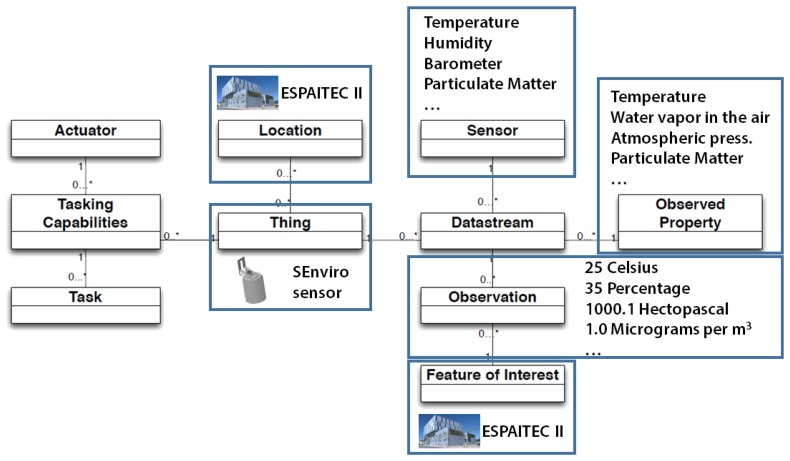
Example of OGC SensorThings API based on a *SEnviro* platform.

Figure 5 shows an example of using OGC SensorThings API for one of the *SEnviro Thing* from the smart campus proof of concept (Section 4). As already discussed, in this standard, the core is the *Things*. Every *Thing* is a *SEnviro Thing* in our *SEnviro* network, which has access with an IP connection; in our case, by Wi-Fi. For this example, we have chosen a sensor located in one of the buildings of our university, called Espaitec II.

Each *Thing* can be associated with one or more locations; in this case, the *Location* is the geographical reference to the place where the sensor is installed. The encoding is performed by GeoJSON [26]. A *Thing* can have many *Datastreams*. A *Datastream* contains the information of a phenomenon. For our use case, we have a *Datastream* for each sensor's phenomenon. Each *Datastream* contains a *Sensor* and an *ObservedProperty*. The first refers to each of the instruments that can observe a phenomenon; in our example, these would be the temperature, humidity, barometer, and so forth. An *ObservedProperty* specifies the phenomenon and also contains the unit of measure. A *Datastream* can have several *Observations*, and they indicate the value for these phenomena. It is encoded by an O&M. In our example, this can be the values taken from a sensor measurement.

Finally, the *FeatureOfInterest* identifies the characteristics of the *Thing*. For our example, this can be the location. Table 2 shows all entities that have been used together with their sensing profile, where each property indicates the type of format that has been encoded. In addition, an example of how it is used is also shown. Currently, OGC SensorThings API is not stable, and it does not have an official implementation. An external server has been used to test this API, which is offered by the OGC SensorThings API staff.

## 4. SEnviro Proof of Concept at Jaume I University Campus

This section presents a proof of concept to test and validate the platform presented in the previous sections. First, the context where the *SEnviro* platform has been deployed is described. In the second subsection, a *SEnviro* network example is developed. The third subsection visualizes the energy consumption of each *SEnviro Thing*. Finally, the web client application developed to access these data is presented and evaluated.

### 4.1. Jaume I University's Context

**Figure 6.**
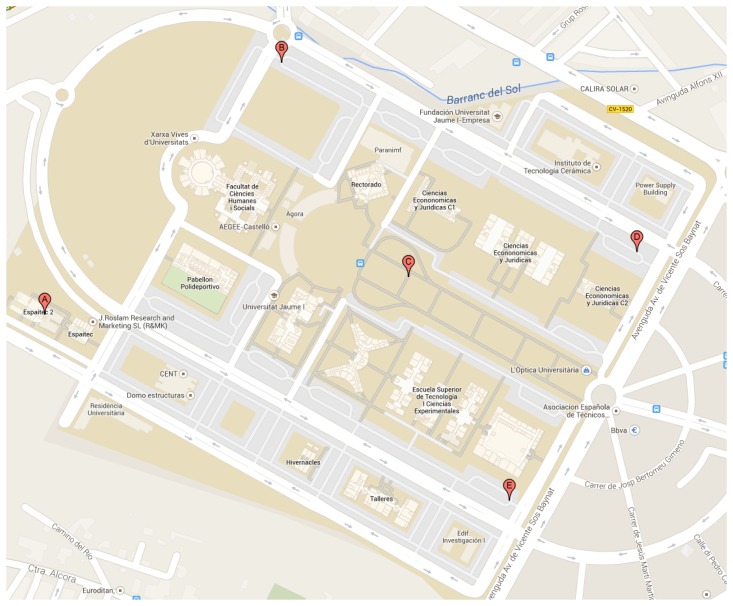
Locations of each *SEnviro Thing* inside the campus of Jaume I University.

The proof of concept has been deployed within the campus at the Jaume I University (http://smart.uji.es/). Moreover, the university campus functions as a small city. As a first step, an environmental monitoring *SEnviro* network has been developed with five different *SEnviro Things*. Figure 6 shows the location of each *SEnviro Thing*.

Each *SEnviro* Thing has been developed with different sensors providing data about some basic phenomena. They are: particles, noise, gases and light. Some of them also include sensors to measure temperature, humidity, atmospheric pressure, rainfall, wind direction and speed. The reason that not all *SEnviro Things* have the same phenomena is that phenomena, such as temperature, humidity, atmospheric pressure, rain, wind speed and direction, do not significantly change within the campus area. To reduce the cost, we have decided to use only two *SEnviro Things* in this network, including all of these sensors.

The created *SEnviro* network for this proof of concept follows the ubiquitous network paradigm [27], where the smart object network is a part of the Internet. Through a gateway, users will have access to the information provided by the smart objects, either directly or through intermediate servers. Usually, a server acts as the sink in the smart object's network, to collect data from each object.

**Figure 7.**
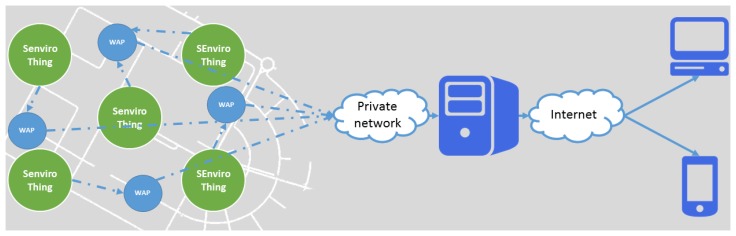
*SEnviro* network for the Jaume I University campus.

Jaume I University has Wi-Fi connection throughout the campus. Each *SEnviro Thing* is connected to the nearest WAP via the included Wi-Fi module and sends the observations to a central server. That server is open to the Internet and is responsible for serving the observations to clients. The configuration chosen for the *SEnviro* network is the direct connection of each *SEnviro Thing* to the WAPs. Therefore, the network is star-shaped (Figure 7), with the particularity that there may be more than one access point.

### 4.2. SEnviro Platform for Environmental Monitoring

An example of the development (hardware and software) of the *SEnviro* platform (presented in Section 3) is shown in this subsection.

#### 4.2.1. Building a SEnviro Thing

**Figure 8.**
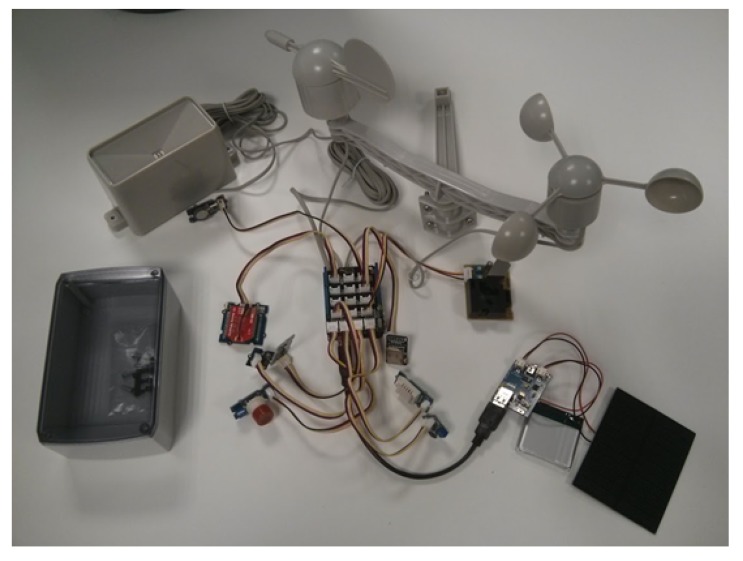
*SEnviro Thing* assembly.

Table 3 shows a general description of the components of a full sensor *SEnviro Thing* regardless of the categories detailed in Section 3.2. The total cost, at the time of writing, per *SEnviro Thing* is 286.28€. Figure 8 shows a *SEnviro Thing* assembled with all components. As commented earlier, each *SEnviro Thing* is composed of five parts (*Core, Sensors, Power Supply, Communication* and *Enclosure*; see Section 3.2). They are described in detail as follows.

**Figure 9.**
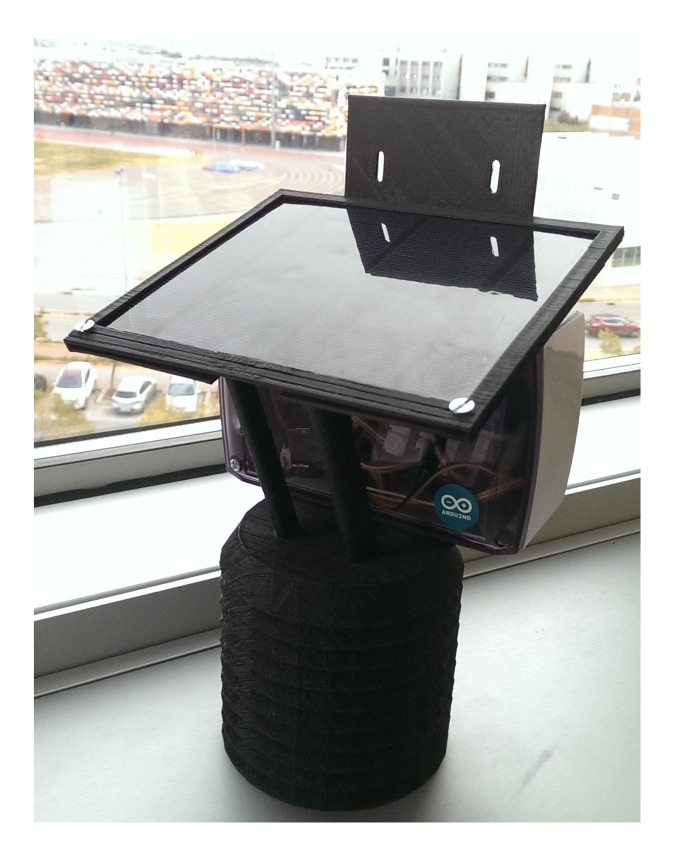
Pagoda box.

- *Core*: The *Core* of the *SEnviro Thing* has four different elements: *Microcontroller, Connectors*, *Clock* and *Memory*. An Arduino UNO [3] has been selected as the *Microcontroller*. As commented in Section 2.2, Arduino UNO offers a low energy consumption and a great number of possibilities of expansion by using shields. These two aspects are critical for this project, because we are looking for an energetically-autonomous platform, and we also want to offer a range of different connections. The Grove shield has been used as the *Connectors*. It has 16 connectors into which Grove elements can be easily plugged. The *Clock* part uses a real-time clock (RTC) Grove module to know the current time. In this way, a timestamp can be attached to each observation when it is sent. The *SEnviro Thing* uses two types of *Memory*. On the one hand, the memory is provided by the Arduino UNO to save the current state of the *SEnviro Thing*. On the other hand, there is external storage through an SD card module to store the observations that have not been successfully sent.
- *Sensors*: Table 4 shows all of the information about the *Sensors* that we have integrated for this study and the characteristics of the measurements that we can obtain from them. All of the *Sensors* that have been chosen are low cost, since one of the goals was to obtain an affordable system. Despite their low price, most of them are suggested by the community to be used for monitoring in industrial environments, so they offer quite reliable measurements. Furthermore, all of the presented sensors have a Grove connector.
- *Communication*: A WiFly RN-XV Wi-Fi module has been used to develop the *Communication* part. It offers this interface in our system allowing the *Core* to send and receive IP packets through a Wi-Fi network. It features Bee socket and connects with the Arduino Universal Asynchronous Receiver-Transmitter (UART) interface via a Bee socket, through its Grove connector. The Grove Bee socket allows the system to replace its communication channel by switching the Bee modules to other compatible Bee communication modules. In this way, we can expand our platform with other kinds of communication, such as mobile data services or Bluetooth.
- *Power Supply*: A lithium battery of 2200 mA has been used to offer an energetically-autonomous platform. It supplies an output voltage of 3.7 V and is charged with the power generated with a 3 W solar panel that supplies an output voltage of 5.2 V. These two elements and the *Microcontroller* are attached to a board (LiPo Rider V1.1 ) that handles the power flow between the various components. This board also has a micro-USB port where the lithium battery can be charged in case solar power is not sufficient. In addition, it does not have to be programmed, as it already comes with an algorithm to manage power sources and drains; nevertheless, it can be replaced with a custom algorithm if needed. Furthermore, a coin cell battery (CR1225) has been used to save the RTC time.
- *Enclosure*: Two enclosure designs have been considered to protect all parts of the *SEnviro Thing*. To protect the *Core, Communication* and *Power Supply* parts, a waterproof enclosure is used; also, a pagoda box (Figure 9) has been designed to deploy the whole assembled system and to protect the Sensors part from weather conditions while still allowing sensors to be in contact with the environment and to provide reliable measurements. The pagoda box has been printed with a 3D printer and is composed of polylactide (PLA), a bio-plastic that is made from corn. While PLA is classified as “industrially compostable”, it is highly UV resistant, much more so than acrylonitrile butadiene styrene (ABS), so it provides a great outdoor housing structure. This box serves as a structure to join all of the parts.

#### 4.2.2. Details of the SEnviro Thing Behavior

An Arduino program has been developed to offer the behavior described in Section 3.3. The Arduino code is written in a file called *sketch*, which is the main program uploaded to the board. Two methods have to be implemented in each *sketch*: *setup* and *loop*. These two methods correspond to the two stages described in Section 3.3. The *setup* method (*initial* stage) is executed as an initializer when Arduino boots up. When setup finishes, the *loop* method (*repetitive* stage) will be executed in an infinite loop.

Arduino does not provide a way to keep track of time and date by default, so we used an external clock. The Arduino requests a reference timestamp from the server at the *setup*, so that timestamps are always up to date when Arduino boots up the first time.

In our system, the Arduino reads the configuration parameters from the server daily. The parameters are the list of active sensors and the frequency of reading of the measurement. This feature allows the behavior of a *SEnviro Thing* to be modified by simply changing the configuration file that is hosted in the centralized server. The changes will be effective in less than 24 h, without the need to deploy a new *sketch* of the Arduino. The response from the server (the configuration parameters) contains some simple instructions, with the tasks that the Arduino should carry out each time it wakes up and their frequency. The response format must be simple, as the processing capabilities of the Arduino are limited. The response is sent in comma separated values (CSV) format.

As said above, the *loop* method executes all of the instructions in an infinite loop. If the behavior of the system indicates a waiting time between runs, the delay function can be used just before ending a *loop* iteration to insert a pause of a desired length. However, this is very expensive in terms of energy consumption, and the batteries would run out of charge very quickly. That is the reason for putting the Arduino to sleep in *SLEEP_MODE_PWR_DOWN* mode, which allows the greatest power savings. To wake up the *Microcontroller*, we use the watch dog timer (WDT) with the largest scale register to trigger an interruption every eight seconds, which is the maximum interval that can be achieved by WDT timer counters. If the system needs longer waiting times, sleep cycles can be counted to check whether the Arduino has to go to sleep immediately after being wakened up or, conversely, has to perform some tasks. The WiFly RN-XV module can also go to sleep by sending a sleep command, and it wakes up whenever it receives data on the RX serial buffer. Thus, the Arduino puts the Wi-Fi module to sleep before going to sleep itself; then, when the interruption is triggered and the Arduino wakes up, it sends a random single character to the Wi-Fi module to wake it up. This wake-up character does not alter the subsequent communication, since it is discarded by the Wi-Fi module.

When the waiting period has finished, our system updates the values of the observations taken from the set of sensors and sends them to the server. However, there could be connectivity problems, so the observation could be lost. To avoid such losses, we have implemented the behavior in the Arduino program to store pending observations in an SD card when sending was not successful. When connectivity is restored, all of those pending observations are sent alongside their corresponding timestamp, and the SD card is cleared. The timestamp is also saved in the SD card for each pending observation to achieve this behavior.

In our approach, many open source libraries have been used to appropriately drive the hardware components. These libraries have been developed by the community and shared on the Internet to facilitate inclusion in other projects with similar requirements, such as ours. Furthermore, we developed our own library for the WiFlyRN-XV module, since we could not find on the web one that suited our needs. Our library is publicly available at Github [28].

### 4.3. Autonomous Power Supply

This subsection introduces the different energy consumptions for each *SEnviro Thing* category and the energy consumption tests with the WDT.

#### 4.3.1. Energy Consumption

Regarding the energy consumption of the Arduino UNO, Table 5 shows a comparison of its power supply requirements in different scenarios. Consumption can vary considerably depending on the peripherals connected to the board.

The energy consumption of the other *Core* components is detailed in Table 6. Both the clock consumptions and the SD card have lower energy consumption than the sensors detailed in the former section. The total energy consumption for these components is 21.5 mA.

Table 7 shows the energy consumption of the *Communication* module depending on its state and activity level. It reveals that it is quite expensive, in terms of energy consumption, to send information; it requires 185 mA. It only consumes 35 mA when it is not transmitting any data, but is ready to receive data. In contrast, it is highly efficient, and it only consumes 4 μA when it is in sleep mode.

The energy consumption of the different *Sensors* is shown in Table 8. This table shows that the energy consumptions of the dust, barometer and gas sensors are significantly higher than the other sensors. The added consumption is 384 mA under normal operating conditions.

#### 4.3.2. Battery Life Tests

As has been commented, the *SEnviro Thing* has been designed to be autonomous, so solar panels and batteries have been used to provide energy. Besides those *Power Supply* elements, it has been necessary to study the energy consumption of the elements of each *SEnviro Thing*. There is no specification from the designers of the Arduino platform about its energy consumption, as it may vary depending on the connected peripherals. In addition to the theoretical aggregated consumption of each *SEnviro Thing*, we have carried out some tests to verify the autonomy and the behavior of the solar panels and batteries.

The theoretical calculations suggest that a battery of 2200 mA should be able to supply energy to the system for four hours and 30 min under a total consumption of 490.5 mA. However, our first autonomy test revealed that each battery could only supply energy to a *SEnviro Thing* for three hours without using any efficiency mechanism. As a conclusion from this first test, it is apparent that energy loses and discharge rates considerably reduce the duration of the batteries. This test was performed without solar panels, to check the battery's capacity.

Furthermore, we considered that it was highly inefficient to keep such consumption rates during the execution time. Therefore, we decided to use WDT interruptions and the sleep mode (see Section 4.2.2) in the Arduino and the Wi-Fi module to: (1) consume as little energy as possible; (2) keep a continuous and steady power supply; and (3) still be able to fulfil the tasks expected to be carried out at each *SEnviro Thing*. The autonomy tests were performed with this improved configuration, and the battery could supply energy to a *SEnviro Thing* for 24 h and 30 min. With this new approach, the batteries lasted eight-times more than under the normal operational mode. The energy consumption obtained is around 90 mA per hour (with energy loses and discharge rates).

These new results are satisfactory, since the critical time for the batteries comes at night when they cannot rely on the energy supplied by solar panels. This represents only half the time of the maximum possible in a day; therefore, the solar panels are capable of recharging the batteries during daylight hours. We are considering the number of solar hours in Spain (min. 9.5 solar hours and max. 14.9 solar hours). For that, we conducted a final test with the batter fully-charged and a 3 W solar panel. The results of this third test were again satisfactory. The energy consumption at night was compensated by the energy supplied from the solar panel during the day.

### 4.4. A Web Client Able to Connect to the SensorThings API

**Figure 10.**
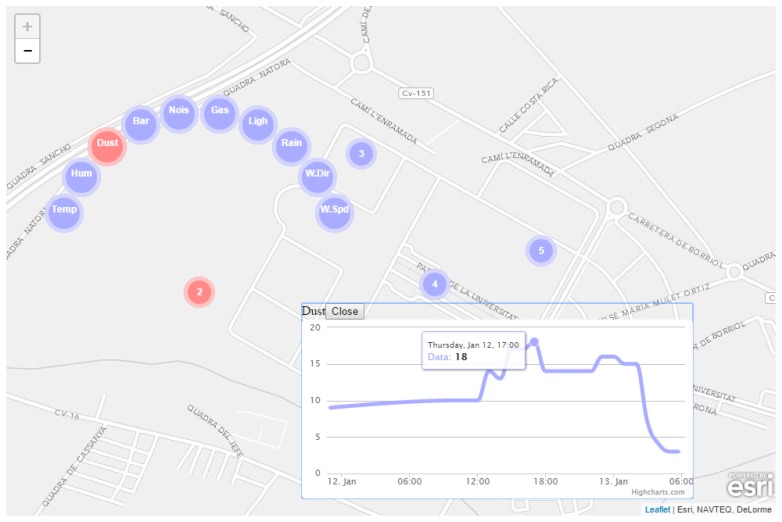
Client developed to show the *SEnviro* network observations.

As a first prototype, a client based on HTML5 [29], JavaScript and Cascading Style Sheets (CSS) has been developed (see Figure 10). This client is able to connect to the OGC SensorThings API service to obtain data from the *SEnviro Things* inside the university. Showing the *Sensors* data in real-time is the main objective of the developed client.

Firstly, the *SEnviro Things* are displayed on a map using markers. When a user clicks on one of them, new markers appear in a pop-up menu. Each new marker symbolizes a sensor corresponding to a data stream of OGC SensorThings API. The selected marker is displayed in red.

When a sensor is clicked on, the client displays a pop-up showing a plot with the latest data for the sensor observations. The plot interactively displays the corresponding observations. It is possible to display different plots simultaneously and even from different sensors provided by multiple *SEnviro Things*. This helps to compare the values of the same phenomenon inside the same network.

To implement the former visualization requirements, we searched for a solution that would offer flexibility, compatibility, as well as standards compliance. There are several frameworks that facilitate creating interactive map clients with HTML5. In particular, we used a combination of already existing frameworks:

- Leaflet (an open-source JavaScript Library for mobile-friendly interactive maps, with ESRI cartography, to put the makers on the map. It proved to be fast and efficient. In addition, it can be executed in restrictive environments, such as smartphones. It is an open-source web mapping library.
- Another library that we used is Bootstrap . It offers the capacity of building a responsive dashboard, and can adapt to the device's features. Furthermore, we use jQuery to handle pop-ups.
- Finally, another framework that was used is Highcharts JS . It is a graphics library written in HTML5 and JavaScript. The library provides an easy and interactive way to generate graphs in a web environment. Highchart JS is free for non-commercial uses.

## 5. Related Work

In the literature, there are some approaches that are similar to our proposal. The following items describe the works that have been analyzed. All of them use open hardware platforms as their basic building blocks.

- The SenseBox project [6] investigates certain real-world use cases for web-enabled sensor objects. The authors present a use case where a motherboard with an Intel atom device connected to an Arduino is used. The Arduino's function consists of connecting the sensors and the motherboard. The objective of the work is to count the traffic load on a road.
- The paper [30] presents a WSN developed using Arduino platforms. A Bluetooth connector is installed in each Arduino. It offers observations for different components, such as CO, CO_2_, temperature and humidity. A worker equipped with a mobile phone connects with the sensor to collect the observations and send them to a server.
- The authors in [31] propose a WSN using Arduino platforms with a ZigBee connection. Each sensor incorporates temperature and humidity sensors. A Raspberry Pi collects the observations and publishes them on the Internet. A web client for visualizing the observations is also presented.
- The authors in [32] present a low-cost solution based on the Arduino platform. Each Arduino is equipped with various sensors, such as CO, CO_2_, hydrogen, methane and sound. In addition, a Global Positioning System (GPS) is also attached to the Arduino. The authors do not specify the wireless connection type.
- The authors in [33] present a sensor prototype using an Arduino Mega board. It takes observations of temperature and humidity on a 5-min interval basis. Although, the device is equipped with a secure digital (SD) card where observations are stored, it can work in real time using an Ethernet connection. It has batteries that last up to 8 h.
- A sensor network built using Arduino platforms is presented in [34]. It is tested using two different network topology configurations: the first configuration uses ZigBee and another one Wi-Fi. In the case of the Wi-Fi configuration, a star topology centralized by a wireless access point (WAP) is used.
- A solution where the users are responsible for collecting data from sensors (crowdsourcing) is presented in [35]. Each sensor provides temperature and humidity data. The system uses the Arduino platform, which can be accessed via Bluetooth. Users can obtain the observations by using the Bluetooth connection in their mobile phones. When a user receives an observation, it is sent to a server, and then all observations are accessible by OGC standards, like SOS. Each observation associates the coordinates of the mobile phone in the instant of the measure.
- The work presented in [36] shows a WSN for monitoring temperature, humidity and soil moisture. Different sensors are connected to a coordinator through ZigBee, which, in turn, is connected to a computer with an Internet connection.
- The authors in [37] present the development of an autonomous sensor using a MSP430 LaunchPad. It is equipped with an accelerometer, light sensor, passive infrared sensor (PIR), temperature sensor and microphone. This system is characterized by having a solar panel that always keeps the battery charged.
- In the last work analyzed [38], a sensor prototype for monitoring agricultural environments is presented. The authors use an Arduino Mega equipped with GPS. Furthermore, it adds a Wi-Fi bridge by means of an Ethernet shield. Different sensors, such as temperature, soil moisture and a light sensor, are attached to the system. This work is especially relevant in the context of our work, because it uses SWE standards, and the WoT and REST paradigms are applied.

In order to compare the formerly reviewed works, Table 9 has a comparison between the detailed works. The following features to characterize each one have been proposed:

- Platform: the microcontroller model that the system uses.
- Real-time: indicates if the system works in real-time. Scale: yes/no.
- Connection: refers to the wireless connections available for the system. Scale: Wi-Fi, Bluetooth, ZigBee, others.
- Phenomena: refers to the phenomena that the system can measure. Scale: temperature, humidity, dust, barometer, noise, others.
- Cost: the cost in terms of money to deploy the system. Scale: Euros.
- IoT-WoT: indicates if the system follows the IoT and WoT paradigms. Scale: yes/no.
- RESTful: shows if the system offers a RESTful interface. Scale: yes/no.
- Client: indicates if the system provides a client to visualize the sensors and observations Scale: yes (what kind)/no.
- OGC standards: shows if the system offers OGC standards. Scale: yes (what kind)/no.

In the following, we compare the analyzed works and our own work using the characteristics listed at the beginning of this section, and the results are shown in Table 9. As can be seen, most of the analyzed works use the Arduino platform, as stated before; this can be explained by its ease of use, the number of different sensors that can be attached to this platform, the large community that supports the Arduino project and its low energy consumption.

Like some other works analyzed, our work offers sensor data in real time. This is possible because all Arduinos are provided with Wi-Fi connection to the Internet. *SEnviro*, in the presented proof of concept, offers different measured phenomena, such as temperature, humidity, dust, barometer, noise, some gases, light, rain gauge and anemometer, being the work offering the largest number of phenomena, among those analyzed.

Regarding cost, our system is less expensive than the other projects; our platform only costs 286.28 €. The platform introduced in [38] is less expensive, but it does not include the sensors. Another characteristic that we want to analyze and include in the comparison is the energy consumption of the system, but only one of the analyzed works [34] offers this information, consuming 268 mA without sensors. SEnviro has a better energy consumption than [34] with 90 mA including all components.

Only two analyzed projects [6,38] follow the IoT and WoT paradigms. *SEnviro* follows these paradigms and offers a RESTful interface. Finally, our project aims to be interoperable, and for this purpose, an OGC standard has been chosen, such as the OGC SensorThings API. Although it is a standard candidate, it has a high probability of being the first OGC standard for the IoT and WoT paradigms.

## 6. Conclusions

This work presents a sensorized platform proposal called *SEnviro*. This concept has been conceived of by following two main objectives: (1) to be developed using open hardware; and (2) to offer interoperability by means of standards. In addition, it is able to work with any kind of sensor.

A platform developed using open-hardware provides many benefits. It offers the possibility to provide access to a large community of developers and facilitates the hardware usage. Furthermore, it enables expansion options, because there are many compatible components, so the schematics are fully available. Another benefit of using open hardware is the low cost of these components, because there are many manufactures that provide the same component.

The other objective is to offer interoperable services to facilitate access to the data. For this aim, the OGC SensorThings API has been used. This API offers an easy and agile access to the sensor data using the IoT and WoT paradigms. The advantage of offering a standard interface is the possibility of reusing the clients.

Our platform offers an easy connection in software and hardware terms. At the hardware level, it offers a plug and play connection using the Grove shield. At the software level, *SEnviro* has developed a *Core*, in which the *Sensors* included in this study can be included with little effort.

The *SEnviro* platform has been designed according the IoT and WoT paradigms. It not only offers a WoT interface, the hardware has been designed taking both paradigms into consideration. Each *SEnviro Thing* can be considered as a smart object permanently connected using the IP protocol.

A challenge for this work was to offer an autonomous *Power Supply*, to avoid any restrictions when deploying the *SEnviro* platform. Another point to note is the sensor and network ability to change its behavior while they are working. This avoids the replacement of the *Sensors* when they are installed and the changing of their settings one by one.

In order to validate the proposed architecture, an environmental sensor network has been created using multiple *SEnviro Things*. A university campus has been chosen as the context for this validation. A network with five *SEnviro Things* has been successfully deployed. Moreover, a web client able to consume data following the OGC SensorThings API provided by the platform has been developed.

As future work, our first objective is to offer a web client that would be able to adjust the *SEnviro Thing* behavior settings. Currently, the settings are provided by means of a simple text file on the server-side. Another line of project development is to add alternative IP-based connectivity, like mobile data services or Bluetooth. Finally, another line of future work is to apply different data analysis to the data provided by the platform, in order to obtain different indicators in the area where the *SEnviro* network has been deployed. These analyses will include the methodologies for real-time event detection applied in previous work [39] in order to detect anomalies in data series from environmental monitoring.

## Figures and Tables

**Table 1.** Comparison between different microcontroller-based platforms.

	***Arduino***	***Raspberry Pi***	***BeagleBone***	***MSP430 Launchpad***
Model	R3	B	A5	1.5
Microprocessor	ATmega328	ARM11	ARMCortex-A8	TI M430G2553
Architecture	8 Bit	32 Bit	32 Bit	16 Bit
Clock speed	16 MHz	700 MHz	700 MHz	16 MHz
RAM	2 KB	256 MB	256 MB	512 B
Flash	32 KB	SD	4 GB	16 KB
Min. power	42 mA	700 mA	700 mA	0.5 uA
Digital input	14	8	66	8
Analog input	6	N/A	7	8
Ethernet	No	Yes	Yes	No
Programming language	Wiring-based	Python, C and Basic	Python, C and more	C/C++
IDE	Arduino tool	IDLE, Scratch, Squeak/Linux	Python,Scratch, Cloud9/Linux	IAREmbedded Workbench Kickstart
Cost	$29.95	$35.00	$199.95	$4.30
Open-hardware	Completely	Partially	Completely	Completely

**Table 2.** Sensing profile properties of OGC SensorThings API used in the *SEnviro Thing* example.

***Entity***	***Property***	***Type***	***Example***
*Thing*	Description	Character String	Espaitec II
*Location*	Time	ISO8601	2014-11-16T10:53:11-0700
*Location*	Geometry	GeoJSON geometry	{“Geometry”: {“type”: “POINT”, “coordinates”: [0, 40]} }
*Datastream*	Description	Character String	Temperature
*ObservedProperty*	URI	URI	urn:ogc:def:property:SEnviro:Temperature
*ObservedProperty*	UnitOfMeasurement	Character String	Celsius
*Observation*	Time	ISO8601	2014-11-16T10:53:11-0700
*Observation*	ResultType	O&M Result Type	Measure
*Observation*	ResultValue	Any (depends on the ResultType)	26.95856250788312
*Sensor*	Metadata	Character String	Espaitec II
*FeatureOfInterest*	Description	Character String	FOI_1
*FeatureOfInterest*	Geometry	GeoJSON geometry	{“Geometry”: {“type”: “POINT”, “coordinates”: [0, 40]}}

**Table 3.** Components list included in the *SEnviro Thing* (prices at the time of writing).

***Category***	***Component***	***Description***	***Cost***
Core	Microcontroller board	Arduino UNO	20.00 €
	Shield Grove	Base Shield V2	8.60€
	Clock	Real-time clock for Grove	5.60€
	MicroSD card module	MicroSD card module for Arduino UNO	8.30€
	MicroSD card	MicroSD card 2 Gb	6.00€
	Screw connectors	Screw terminal for Grove	2.75€
	Box for arduino	RETEX series 102	10.65 €
	Box for sensors	3D printed box	10.00 €

Communication	Wi-Fi module	RN-XV WiFly module	40.80 €
	Socket Bee	Bee socket for Grove	6.72€

Sensors	Temperature and humidity sensor	Grove temperature and humidity sensor	12.60 €
	Loudness sensor	Grove noise sensor	4.95€
	Light sensor	Grove light sensor	2.90€
	Dust sensor	Grove particulate matter sensor	14.45 €
	Barometer sensor	Grove barometer sensor	16.35 €
	Gas sensor	Grove MQ-9 sensor	7.95€
	Rainfall, wind speed and direction sensors	Weather meters	72.00 €

Power supply	Power module	LiPo Rider	9.50€
	Battery	Polymer lithium ion battery 2200 mA 3.7 V	9.66€
	Solar panel	3 Wsolar panel 138 × 160	15.00 €
	Coin cell battery	CR1225 3 V 12 mm 47 mA coin cell battery	1.50€

**Table 4.** Details of the included Sensors.

***Sensor***	***Phenomena***	***Manufacturer***	***Model***	***Data Interface***	***Units***	***Range***	***Accuracy***
DHT22	Temperature Humidity	Seedstudio	SEN51035P	Analog	Centigrade Rate	[−40, 80] [5%, 99%]	±0.5 Degrees (C) ±2 RH
Bosch BMP085	Pressure Temperature	Seedstudio	SEN05291P	I2C	Hectopascal Centigrade	[300, 1100] [−40, 85]	±0.03 hPa ±2 gradosC
LDR GL5528	Light intensity	Seedstudio	SEN11302P	Analog	Lux	[0, 1024]	Not specified
LM2904 Amplifier	Loudness	Seedstudio	SEN02281P	Analog	Decibel	[0, 1024]	Not specified
PPD42NS	Dust/particles	Seedstudio	SEN12291P	Digital	pcs/liter	[0,28,000]	>1 um
MQ-9	CO Combustible gas	Seedstudio	SEN04092P	Analog	ppm ppm	[10, 1000] [100, 10,000]	Not specified Not specified
Weather meters	Wind speed Wind direction Rain meter	Sparkfun	SEN08942	Analog (RJ11)	km/h Direction (degrees) mm	Not specified [0,360] Not specified	Not specified Not specified Not specified

**Table 5.** Arduino UNOenergy consumption.

**Mode**	**Energy Consumption (mA)**
Sleep	5–9
Normal	25–50
High Power	300

**Table 6.** Energy consumption of the *Core* components.

**Component**	**Energy Consumption (mA)**
Clock	1.5
MicroSD card module	20

**Table 7.** WiFly RN-XV energy consumption.

**Mode**	**Energy Consumption (mA)**
Sleep	4 × 10^−3^
RX active	35
TX active	185

**Table 8.** *Sensors* energy consumption.

**Component**	**Energy Consumption (mA)**
Temperature and humidity sensor	1.5
Loudness sensor	0.5
Light sensor	3
Dust sensor	90
Barometer sensor	89
Gas sensor	150
Rainfall, wind speed and direction sensors	50

**Table 9.** Comparison between different sensorized platforms.

**Reference**	***Platform***	***Real-Time***	***Connection***	***Phenomena***	***Cost***	***IoT-WoT***	***RESTful***	***Client***	***OGC Standards***
[6]	Arduino and Intel Atom	Yes	UMTS-3G USB	N/A (N/A: Not specified)	375€ (without sensors)	Yes	No	No	SWE
[30]	Arduino	No	Bluetooth	CO, CO_2_, Temperature and Humidity	N/A	No	No	Google Web Toolkit	No
[31]	Arduino and Raspberry	Yes	ZigBee/Ethernet	Temperature and Humidity	N/A	No	No	HTML	No
[32]	Arduino	Yes	N/A	CO, CO_2_, Hydrogen, Methane and Noise	N/A	No	No	HTML	No
[33]	Arduino Mega	No	Ethernet	Temperature and Humidity	N/A	No	No	Drupal	No
[34]	Arduino UNO	Yes	Wi-Fi	Temperature, Humidity, Barometer and Gases	820€	No	No	No	No
[35]	Arduino UNO	Yes	Bluetooth	Temperature and Humidity	N/A	No	No	Android	SOS
[36]	Arduino UNO	Yes	ZigBee	Temperature, Humidity and Soil Humidity	N/A	No	No	No	No
[37]	Texas Inst. MSP430	N/A	Not specified	Temperature, Humidity, PIR, Noise and Accel.	N/A	No	No	No	No
[38]	Arduino Mega	Yes	Ethernet	Temperature, Soil Humidity and Light	185€	Yes	No	No	O&M
Current work	Arduino UNO	Yes	Wi-Fi	Temperature, Humidity, Dust, Barometer, Noise, Gases, Light, Rain Gauge and Anemometer	286.28 €	Yes	Yes	HTML5	OGC SensorThings API

## References

[1] ONeill R.V., Hunsaker C.T., Jones K.B., Riitters K.H., Wickham J.D., Schwartz P.M., Goodman I.A., Jackson B.L., Baillargeon W.S. (1997). Monitoring environmental quality at the landscape scale. BioScience.

[2] Kortuem G., Kawsar F., Fitton D., Sundramoorthy V. (2010). Smart objects as building blocks for the Internet of things. IEEE Internet Comput..

[3] Official Arduino Website http://www.arduino.cc.

[4] Rodrigues J.J.P.C., Neves P.A.C.S. (2010). A survey on IP-based wireless sensor network solutions. Int. J. Commun. Syst..

[5] Uckelmann D., Harrison M., Michahelles F. (2011). An Architectural Approach towards the Future Internet of Things. Architecting the Internet of Things.

[6] Bröring A., Remke A., Lasnia D. (2012). SenseBox–A Generic Sensor Platform for the Web of Things. Mobile and Ubiquitous Systems: Computing, Networking, and Services.

[7] Guinard D., Trifa V., Pham T., Liechti O. Towards Physical Mashups in the Web of Things.

[8] OGC SensorThing API http://ogc-iot.github.io/ogc-iot-api.

[9] Sohraby K., Minoli D., Znati T. (2007). Wireless Sensor Networks: Technology, Protocols, and Applications.

[10] Liu D., Ning P. Establishing Pairwise Keys in Distributed Sensor Networks.

[11] Faludi R. (2010). Building Wireless Sensor Networks: With ZigBee, XBee, Arduino, and Processing.

[12] Alliance Z. ZigBee Home Automation Public Application Profile, Revision 15. http://docs.zigbee.org/zigbee-docs/dcn/07/docs-07-5367-02-0afg-home-automation-profile-for-public-download.pdf.

[13] Z-Wave Z-WaveProtocol Overview Revision 4. http://wiki.ase.tut.fi/course.

[14] Darbee P. INSTEON: The Details. http://www.insteon.net/pdf/.

[15] Zorzi M., Gluhak A., Lange S., Bassi A. (2010). From today's INTRAnet of things to a future INTERnet of things: A wireless- and mobility-related view. IEEE Wirel. Commun..

[16] Buratti C., Conti A., Dardari D., Verdone R. (2009). An Overview on Wireless Sensor Networks Technology and Evolution. Sensors.

[17] Yazar D., Dunkels A. Efficient Application Integration in IP-based Sensor Networks.

[18] Fielding R.T., Taylor R.N. (2002). Principled Design of the Modern Web Architecture. ACM Trans. Internet Technol..

[19] Pearce J.M. (2012). Building Research Equipment with Free, Open-Source Hardware. Science.

[20] Official RaspBerry Pi Website http://www.raspberrypi.org.

[21] Official BeagleBoard Website http://beagleboard.org.

[22] Official MSP430 Launchpad Website http://www.ti.com/launchpad.

[23] Sheth A., Henson C., Sahoo S.S. (2008). Semantic Sensor Web. IEEE Internet Comput..

[24] Medagliani P., Leguay J., Duda A., Rousseau F., Duquennoy S., Raza S., Ferrari G., Gonizzi P., Cirani S., Veltri L. (2014). Volume Bringing IP to Low-power Smart Objects: The Smart Parking Case in the CALIPSO Project. Internet of Things Applications— From Research and Innovation to Market Deployment.

[25] Tamayo A., Viciano P., Granell C., Huerta J. (2011). Empirical Study of Sensor Observation Services Server Instances. Advancing Geoinformation Science for a Changing World.

[26] GeoJSONA http://geojson.org.

[27] Puccinelli D., Haenggi M. (2005). Wireless sensor networks: Applications and challenges of ubiquitous sensing. IEEE Circuits Syst. Mag..

[28] WiFlyRN-XV library https://github.com/alujans/WiFlyRNXV.

[29] Hyatt D., Hickson I. HTML 5. W3c working draft, W3C, 2008. http://www.w3.org/TR/2008/WD-html5-20080610/.

[30] Mendez D., Perez A., Labrador M., Marron J. P-Sense: A participatory sensing system for air pollution monitoring and control.

[31] Ferdoush S., Li X. (2014). Wireless Sensor Network System Design Using Raspberry Pi and Arduino for Environmental Monitoring Applications. Procedia Comput. Sci..

[32] Abraham K., Pandian S. A Low-Cost Mobile Urban Environmental Monitoring System.

[33] Baker E. (2014). Open source data logger for low-cost environmental monitoring 2014. Biodivers. data j..

[34] Dines E., Al-Majeed H., Fernando A., Abdalla M., Gohil J. A new WSN paradigm for environmental monitoring and data collection.

[35] Davidovic N.D.R., Stoimenov L. Ardsense: Extending mobile phone sensing capabilities using open source hardware for new citizens as sensors based applications.

[36] Gaddam A., Al-Hrooby M., Esmael W. Designing a Wireless Sensors Network for Monitoring and Predicting Droughts.

[37] Voigt T., Ritter H., Schiller J., Conti M., Giordano S., Gregori E., Olariu S. (2003). Solar-Aware Routing in Wireless Sensor Networks. Personal Wireless Communications.

[38] Demuth D. (2012). A Web of Things integrated Sensor Platform for Precision Agriculture. Bachelor's Thesis.

[39] Trilles S., Schade S., Belmonte O., Huerta J. Real-time anomaly detection from environmental data streams.

